# *Entamoeba histolytica* and amoebic liver abscess in northern Sri Lanka: a public health problem

**DOI:** 10.1186/s41182-020-0193-2

**Published:** 2020-01-22

**Authors:** Tharmegan Tharmaratnam, Thirunavukarasu Kumanan, Mina Amin Iskandar, Katrina D’Urzo, Prasaanthan Gopee-Ramanan, Mayura Loganathan, Tyler Tabobondung, Taylor Anthony Tabobondung, Seyon Sivagurunathan, Mitul Patel, Iqdam Tobbia

**Affiliations:** 1 School of Medicine, Royal College of Surgeons in Ireland, Dublin, Ireland; 2 School of Medicine, Royal College of Surgeons in Ireland-Bahrain, Busaiteen, Bahrain; 3 Department of Internal Medicine, Faculty of Medicine, University of Jaffna, Jaffna, Sri Lanka; 4 Teaching Hospital Jaffna, Faculty of Medicine, University of Jaffna, Jaffna, Sri Lanka; 5 Department of Diagnostic Radiology, Hamilton Health Sciences Centre, Hamilton, ON Canada; 6 Department of Radiology, Michael G. DeGroote School of Medicine, McMaster University, Hamilton, ON Canada; 7 Academic Family Health Team, Mount Sinai Hospital, Faculty of Medicine, University of Toronto, Toronto, ON Canada; 8 Department of Family and Community Medicine, Faculty of Medicine, University of Toronto, Toronto, ON Canada; 9 Department of Family Medicine, Brantford General Hospital, Hamilton, ON Canada; 10 Department of Family Medicine, Michael G. DeGroote School of Medicine, McMaster University, Hamilton, ON Canada; 11 Department of Physical and Environmental Sciences, University of Toronto, Toronto, ON Canada; 12 Stonechurch Family Health Clinic, Department of Family Medicine, Michael G. DeGroote School of Medicine, McMaster University, Hamilton, ON Canada; 13 Department of Pathology and Clinical Microbiology, School of Medicine, Royal College of Surgeons in Ireland-Bahrain, Busaiteen, Bahrain

**Keywords:** *Entamoeba histolytica*, Amoebic liver abscess, Sri Lanka, Protozoan

## Abstract

*Entamoeba histolytica* (*E. histolytica*) is a facultative protozoan parasite implicated in amoebic liver abscesses (ALA), the most common extraintestinal manifestation of this infection. *E. histolytica* is endemic to sub-tropical and tropical countries and has been a major public health concern in northern Sri Lanka (SLK) for the last three decades. This has been attributed to a multitude of factors such as poor sanitation, hygiene, male sex, middle age, overcrowding, unsanitary practices in the production of indigenous alcoholic beverages, and alcohol consumption. Additionally, while rates of *E. histolytica* have declined substantially throughout the rest of the island, largely due to better infrastructure, it remains pervasive in the northern peninsula, which is generally less developed. Infection arises primarily from fecal-oral transmission through the consumption of contaminated drinking water containing cysts. Upon ingestion, cysts multiply into trophozoites and colonize the host colonic mucosa using lectin and cysteine proteases as virulence factors, leading to host invasion. Symptoms occur along a spectrum, from asymptomatology, to pyrexia, abdominal cramping, and amoebic dysentery. Colonization of the colon results in the formation of distinct flask-shaped ulcers along the epithelium, and eventual penetration of the lamina propria via the production of matrix metalloproteinases. ALA then develops through trophozoite migration via the mesenteric hepatic portal circulation, where microabscesses coalesce to form a single, large right-lobe abscess, commonly on the posterior aspect. The progression of infection to invasive disease is contingent on the unique interplay between host and pathogen factors, such as the strength of host-immunity to overcome infection and inherent pathogenicity of the *Entamoeba* species. As a preventable illness, *E. histolytica* complications such as ALA impose a significant burden on the healthcare system. This mini-review highlights epidemiological trends, risk factors, diagnostic modalities, treatment approaches, and opportunities for prevention of *E. histolytica*-induced ALA, to help address this endemic problem on the island of SLK.

## Introduction

*Entamoeba histolytica* (*E. histolytica*) is a non-flagellated, facultative protozoan enteropathogen which affects 50 million individuals globally [1]. It is the fourth leading cause of mortality worldwide due to parasitic infection, and in 2013 was responsible for 11,300 deaths across the globe [2]. In addition to being a significant cause of protozoal diarrhea and dysentery, *E. histolytica* is also the most common cause of amoebic abscesses [3]*.* This pathogen is endemic to tropical countries, such as Sri Lanka (SLK) and has been a major parasitological health concern on the island since 1962 [4–9]. Overall, the incidence of amoebiasis and ALA have declined significantly in other parts of the island [10] but remains a common cause of emergency department (ED) admission in the north [6, 11]. This has been attributed to a multitude of factors such as poor sanitation, hygiene practices, alcohol consumption, male sex, low socioeconomic status (SES), lack of safe water, healthcare facilities and poor access to healthcare services [12, 13]. Opportunities to completely eradicate this pathogen from the island have been circumvented by political unrest and internal displacement, which took place in the northern regions [11, 14]. With the unrest’s resolution in 2009, research has once again begun in a concerted effort to eradicate this infection from the island*.* Thus, preventive medicine is crucial and may contribute to reducing the incidence of illness through the implementation of health policy initiatives and control strategies. This mini-review highlights epidemiological trends, risk factors, diagnostic modalities, treatment approaches, and prevention strategies to reduce transmission and the burden of *E. histolytica* in SLK.

## Epidemiology

*Entamoeba* infection can be divided into symptomatic and asymptomatic. Asymptomatic individuals tend to be infected/colonized by mostly non-pathogenic and commensal *Entamoeba* species, primarily *Entamoeba dispar* (*E. dispar*), rather than the pathogenic *E. histolytica* [15–17]. In fact, Herbinger et al. (2011) demonstrated that, in a cohort of 5378 travelers returning back to Germany from elsewhere with symptoms of intestinal infections, PCR had detected *E. histolytica* and *E. dispar* in 9.7% and 88.3% of cases, respectively; more importantly, however, was the fact that almost all of those with PCR evidence of *E. histolytica* had symptoms typical of amebiasis, compared with only half with evidence of *E. dispar* [18]. The rest of those with *E. dispar* were determined to be co-infected with other organisms such as *Campylobacter* spp., *Giardia lamblia*, and *Salmonella typhi*. Moreover, some studies have even showed *E. dispar* colonization in completely asymptomatic HIV-positive individuals, with no evidence of mucosal invasion [15, 19].

Up until recently, microscopy has been the most widely used method of diagnosis in studies and in practice [20]. However, microscopy is vastly user-dependent and (unless the user is highly skilled and experienced) lacks ability in diagnosing true amebiasis from amebiasis-like symptoms caused by other microorganisms, but with concomitant *E. dispar* colonization [21]. As such, going forward with this discussion, it is important that a discrepancy is maintained between the epidemiology of *Entamoeba* infection (which may be completely asymptomatic) and the epidemiology of the disease itself (amebiasis and its many manifestations, caused primarily by *E. histolytica*).

The World Health Organization (WHO) estimates that around 500 million individuals are infected with *Entamoeba* spp.; 50 million of those with an invasive disease such as ALA [22]. Other accounts claim that *E. histolytica* afflicts 10% of the population worldwide [23], with an estimated death toll of 40,000–100,000 per year, making it the second most common cause of mortality from an infectious parasitic disease [24]. A recent review by Cui et al. (2019) assessing articles that had used molecular methods (PCR) for detection uncovered that, of the 107,396 total pool of participants of all included studies worldwide, 3817 (3.55%) were positive for *Entamoeba* spp.; rates were as low as 0.43% in Belgium and as high as 82.64% in Malaysia [22]. It is worth mentioning that, although some studies conducted in developed countries may show high PCR-positivity rates for *Entamoeba* spp., the proportion of those positive for the virulent *E. histolytica* is minor. For example, out of the 66 patients positive for *Entamoeba* spp. in a small-scale Canadian study, only 2 (0.03%) were positive *for E. histolytica* [25]. Similar results are apparent for other studies in other developed countries such as Germany (0.1%), Sweden (0.06%), and Australia (0.04%) [18, 20, 26, 27]. In contrast, the proportion of PCR-identified *E. histolytica* infections compared with total *Entamoeba* infections in developing countries neighboring SLK like India, Bangladesh, Pakistan, and Malaysia were 41.4%, 60.2%, 17.2%, and 28.1%, respectively [28–43]. Therefore, the literature largely suggests that most infections by *E. histolytica* occur in developing countries, and those from developed countries that are infected are often returning travelers or immigrants from endemic areas [44]. The burden of disease in developing nations like SLK is primarily due to low SES, inadequate sanitation, and unhygienic practices [13, 45]. In these areas, symptomatic *E. histolytica* infections are a significant cause of morbidity and mortality, with deaths largely being attributed to complications of infection, such as extra-intestinal manifestations (i.e., ALA) [46].

The first published report of *E. histolytica* in Sri Lanka dates back to 1962 [7]. Since then, numerous studies have highlighted the burden of this disease as a major public health concern [6, 7, 9, 11, 47]. In 1985, ALA accounted for 5.9% of total admissions at the Teaching Hospital Jaffna (THJ) [4]. In 2015, it was reported to be 3.0 per 10,000 admissions at THJ, highlighting that this region of the island is still largely endemic to *E. histolytica* [6]. In a longitudinal study, Kannathasan et al. reported 346 confirmed ALA cases in northern SLK over a 3-year period, with 99.7% of serum samples testing positive for the IgG antibody against *E. histolytica* [48]. While no current data exists on other parts of the island, an early study by Rajasuriya and Nagaratnam (1962) reported 1.5% of total admissions at the General Hospital Colombo, the capital city, are due to ALA [7]. A more recent report by Kannathasan et al. (2018) states that ALA is seldom seen in other parts of the island compared with northern SLK [6].

## Pathogenesis of *Entamoeba histolytica*

Relative to other enteropathogens, *E. histolytica* has a simple life-cycle. Infection begins when humans, the solitary natural hosts, ingest quadrinucleated cysts found in fecally contaminated water or food [45, 49]. It can also occur through swimming in contaminated bodies of water, exposure in endemic areas, and also through person-to-person contact [50, 51]. Once ingested, cysts transit through the gastrointestinal system into the small bowel, and can then migrate to the large intestine. *E. histolytica* cysts undergo excystation, which releases trophozoites (10–20 μm) in the lumen of the intestinal wall, that reproduce via binary fission. The trophozoites then penetrate the colonic mucosa, forming distinct flask-shaped ulcers [50]. The trophozoites can then gain access to the hepatic portal circulation, resulting in hematogenous spread to the liver, which produces an inflammatory reaction leading to necrotic hepatocytes and subsequent abscess formation with a characteristic ‘anchovy-paste’ exudate [46].

**Fig. 1:**
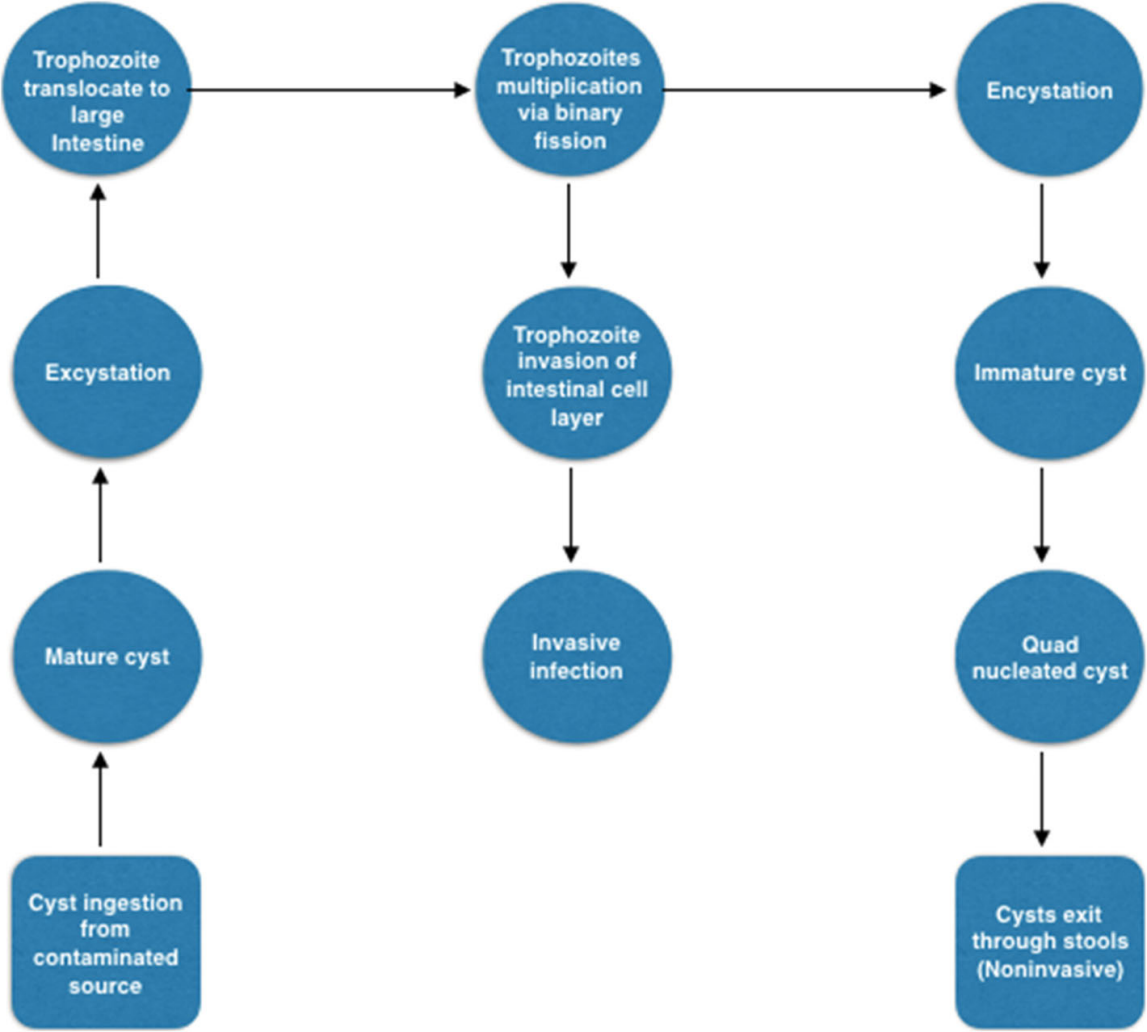
Schematic representation of the pathogenesis of *E. histolytica* from ingestion to extra-intestinal hepatic invasion

Ultimately, the progression to symptomatic disease is dependent on a multitude of factors including gut microbiome composition, dysbiosis, hosts’ age, hosts’ genetic composition, immunogenicity (i.e., reduced cell-mediated immunity), and the unique interaction between host-immunity and parasitic virulence. These have been shown to be a predictor of disease severity [9, 52–55], and are discussed in Fig. 1 below.

## *Entamoeba histolytica*-related virulence factors

*E. histolytica* contains several virulence factors, which contribute to its pathogenicity and invasiveness. The most identified virulence factor is the galactose and *N*-acetyl-d-galactosamine (Gal/GalNAc) specific lectin, which is responsible for the ability of *E. histolytica* to adhere to mucosal cells in the bowel. This occurs by targeting the *O*-linked polysaccharide side chains of mucin on colonic epithelial cells [54].

While prostaglandin E2 (PGE_2_) functions in impairing macrophage function, it is also known to cause mucus hypersecretion of intraepithelial cells and eventual mucus depletion. Mucus hypersecretion occurs as a host defense mechanism to repel the parasite from adhering to the colonic wall [56]. PGE_2_ is also known to disrupt tight junction integrity of epithelial cells allowing parasitic infiltration [54, 57], thus increasing gut permeability. This results in chloride secretion into the intestinal lumen, which eventually manifests as amoebic diarrhea [56]. *E. histolytica* also exerts its pathogenicity through induction of apoptosis of host cells, in addition to a relatively new mechanism of cell-destruction called ‘trogocytosis’ (ingestion of small constituents of host cells, prior to induction of apoptosis) [58, 59].

Another virulence factor contributing to the colonic mucosal barrier breach in *E. histolytica* trophozoites are cysteine proteases (CP), which are hydrolytic enzymes that participate in the destruction of host epithelial and inflammatory cells and subsequent invasion. In *E. histolytica*, CP-1, CP-2, and CP-5 account for 90% of all cysteine protease activities during infection, with CP-5 being the most predominant in degradation of the epithelial mucus layer [56]. CP-5 secretion induces cleavage of C-terminus of the MUC2 protein, which leads to degradation of the protective mucin barrier, and subsequent invasion and increased gut hyperpermeability. Peroxiredoxin, alcohol dehydrogenase, and lipopeptidophosphoglycan are other virulence factors which are implicated in the evasion of host defenses by *E. histolytica* [60].

Ultimately, invasive diseases of the colonic mucosa require disruptions in the extracellular matrix (ECM) for progression into the lamina propria. *E. histolytica* produces *E. histolytica* migration inhibitory factor (EhMIF), which causes mucosal inflammation and downstream production of matrix metalloproteinases (MMPs), leading to ECM degradation and invasion [58], thus EhMIF further contributes to disseminated disease. Trophozoites enter the liver hematogenously through the hepatic circulation, forming microabscesses that eventually amalgamate to form a well-circumscribed ALA, generally in the posterior right lobe [61]. The abscess generally consists of inflammatory debris, dead hepatocytes, and amoebic trophozoites surrounded by a rim of connective tissue, with a characteristic “anchovy paste” exudate [62, 63].

## Risk factors

Several risk factors are involved in the development of ALA via invasion of *E. histolytica* into the hepatic circulation. These risk factors are discussed in detail below.

### Alcohol consumption

Indigenous alcohol consumption, particularly from the fermented sap of the Palmyra toddy (*Borassus falbellifer*) is a well-known cause of ALA in SLK [13], such correlation has previously been demonstrated in several large population-based studies [3, 6, 64]. An early study by Hai et al. (1991) demonstrated that alcohol consumption was implicated in 85% of 220 confirmed ALA cases [65]. In another study of 50 confirmed ALA cases over a 15-month period, Fernando et al. (2011) reported 96% of ALA cases were attributable to Palmyra toddy consumption [6], a practice widely associated with lower socioeconomic background (i.e., labourers) within taverns where hygienic conditions are often poor [66]. Kannathasan et al. (2014) assessed knowledge and attitudes regarding ALA in a cohort of 90 patients admitted to THJ for ALA. They concluded 80% to be regular alcohol consumers, with 90% under the belief that alcohol had no detrimental impact on the liver [67]. In a more recent longitudinal study of 367 ALA patients between 2012 and 2015, Kannathasan et al. (2018) demonstrated alcohol consumption to be an independent risk factor for ALA (OR 4.5; 95% CI 1.94–11.96) [6]. Additionally, almost all patients were males (largely between 31 and 50 years of age) with a history of alcohol consumption. Interestingly, the highest consumption occurred during the dry season, which correlated to peak alcohol sales in the northern region [7, 10]. Currently, data supports an association between the consumption of indigenous alcoholic beverages and ALA [64, 68]. While it is unclear how *E. histolytica* enters the alcoholic beverages prior to consumption to cause disease, theories suggest that it may be due to factors such as water contamination, unhygienic practices in toddy taverns such as open-air defecation, improper hand hygiene, and water filtration practices (cysts are resistant to low doses of chlorination) [9, 69].

Numerous mechanisms have been postulated to explain the link between alcohol consumption and the development of ALA. One such explanation is the ‘iron hypothesis’, whereby iron sequestration in the liver leads to overload, preventing alcohol metabolism via aldehyde dehydrogenase, requiring iron as a co-factor [68]. Others have suggested the role of alcohol-induced hepatocyte injury to result in reduced immunity, and thus increased susceptibility to infection due to damage of Kupffer cells [64]. In vitro studies have demonstrated that the presence of iron promotes the growth of amoeba, while also contributing to its virulence and cytotoxicity [70], which generally becomes elevated with indigenous alcohol consumption. Indeed, the constituents of toddy include alcohol, as well as iron (11.01 mg of iron/100 g of toddy) [9, 66]. While it can only be speculated that iron content in the liver is an important factor in the pathogenesis of ALA, the exact mechanisms remain yet to be elucidated [71]. A proposed mechanism involves the role of chronic alcohol consumption as a contributing factor to ALA development. Specifically, alcohol is known to elicit suppression of circulating hepcidin in the liver, ultimately resulting in an increased number of divalent metal transporter-1 in the enterocytes of the duodenum [72]. The role of iron metabolism may partly explain the lower incidence of ALA amongst females. Indeed, Wuerz and colleagues (2012) suggest that iron deficiency anemia and protective hormonal factors in women of reproductive age as potential explanations for the lower observed ALA rates in females [44].

Alcohol consumption as a risk factor for *E. histolytica* is further exacerbated by increased rates of alcohol consumption on the island, which impose a significant economic burden on the healthcare system [10]. The economic cost of alcohol-related health conditions in SLK accounts for 1.07% of the GDP (885.7 million USD), with direct costs to alcohol-associated conditions estimated to be 388.3 million USD [73]. Importantly, the WHO reports 5.6% of the population in SLK to have alcoholism, which is greater than the entire Southeast Asia region (3.0%) [74]. Rates of alcoholism coupled with poor sanitation in toddy taverns and inadequate personal hygiene provide the perfect milieu for *E. histolytica* to develop. Indeed, Mukhopadhyay et al. (2010) demonstrated patients with alcoholism generally have ALA of greater size, coupled with delayed resolution and greater frequency of complications [3].

### Socioeconomic status and poor hygiene

Poor sanitary conditions coupled with unhygienic practices are common amongst individuals of low SES and provide the optimal environment for the development of ALA in SLK [6, 11, 40]. An early study in neighboring India demonstrated 67.5% of ALA patients to be of low SES, with 72% afflicted with high alcohol consumption [64]. A more recent study by Kumanan et al. (2018) demonstrated the majority of ALA cases in SLK arise in from families of lower SES, such as manual laborers and farmers, where good hygiene often cannot be expected [9]**.** In another study by Kannathasan et al. (2018) from the northern region, 97% of ALA patients drank toddy, and over 80% obtained their drinking water from unprotected wells; 88% drank toddy at taverns; 80% practiced open-air defecation at the taverns [6].

SES may also be correlated to geographical location. In fact, Kannathasan et al. (2018) demonstrated that 75% of patients diagnosed with ALA originated from the rural regions of SLK [6]. This may be associated with a lack of infrastructure and healthcare disparities in these regions, which could contribute to the burden of disease. Indeed, Wuerz and colleagues (2012) suggest SES to be a risk factor for invasive amebiasis, including ALA [44].

### Male sex

ALA generally affects men between 18 and 50 years of age, with the greatest prevalence occurring between the 4th and 5th decades of life [18, 42]. The prevalence is also estimated to be 7–10 times greater in males compared with females [52, 53]. Singh et al. (2019) demonstrated that amongst 115 ALA cases in India, 93% were reported in males. These patients were four times more likely to have a habit of alcohol consumption (OR 4.0; 95% CI 1.2–13; *p* = 0.019) [46]. Kannathasan et al. (2018) also demonstrated similar findings amongst a cohort of ALA patients, in which all were found to male, with a history of heavy alcohol consumption [6].

While the predilection to preferentially affect males than females is not entirely clear, several mechanisms have been postulated. These include increased alcohol consumption leading to elevated hepatocellular damage amongst males compared with females [74], which may predispose them to an increased risk of infection. Animal models have also suggested testosterone to favor the development of ALA. Cervantes-Rebolledo et al. (2009) demonstrated ALA to be absent in 50% of gonadectomized male hamsters, in comparison to an infection rate of 100% after intraportal administration of trophozoites in non-gonadectomized controls. Assays also demonstrated scarce inflammatory infiltrate and areas of necrosis in gonadectomized hamsters, with a Th2 and Th3 cytokine profile, while non-gonadectomized samples showed a marked Th1 inflammatory cytokine response, suggesting a downregulation of the inflammatory response in gonadectomized models [75]. Another explanation for male-predominance may be related to the aforementioned ‘iron hypothesis’. Other factors that can explain the increased prevalence in males is the fact that males in SLK drink more; indeed, a study by the WHO demonstrated the alcohol consumption rate amongst females to be 6.4%, while it was found to be almost nine times more common amongst males (53.1%) [76].

## Clinical manifestations

ALA is the most common extraintestinal manifestation of *E. histolytica* [77]. However, 90% of infected patients remain asymptomatic, while only 10–20% become symptomatic [49]. The two most common presenting complaints of ALA in symptomatic patients are right hypochondriac pain, and fever (38.5 to 39.5 °C), which generally presents within 2–4 weeks in 50–80% of individuals [6, 13, 44, 52]. Patients may also present with nausea, vomiting, weakness, weight loss, and referred pain to the shoulder in some cases. Patients may or may not present with jaundice [78].

Generally, simultaneous colonic infection is seen in 50% of patients, presenting with ulcers commonly near the ileocecal valve and cecum [13]. Other complications include acute fulminant necrotizing amoebic colitis, toxic megacolon, toxic myocarditis, right iliac fossa mass, and acute appendicitis [47, 79–82]. Recent work also suggests increased co-infection rates of entamoeba with concomitant pulmonary tuberculosis [83], as well as a greater prevalence of rates of infection in patients with inflammatory bowel disease (Crohn’s disease or ulcerative colitis), compared with the general population [84].

Another rare complication is cardiac infection due to ALA rupture and spread to the pericardium, which can cause intrapericardial rupture and resultant cardiac tamponade, or slow-onset pericardial effusion. However, this generally occurs if the abscess is found in the left lobe of the liver, which is rare, as most cases affect the posterior right lobe [85]. Hematogenous spread to the brain can also occur, resulting in cerebral amoebiasis, but is exceedingly rare [86]. Finally, the risk of recurrent infection, despite adequate treatment has also been previously described in the literature [87].

Additionally, distinguishing ALA from pyogenic liver abscesses (PLA) is crucial due to the differential approach to management in both conditions (see Table 1), despite similar clinical presentation (pyrexia, RUQ pain, hepatomegaly) [48]. While clinical findings alone may be insufficient to distinguish between PLA and ALA [93], certain clinical features can aid in differentiating the two. For example, generally, PLA is polymicrobial (commonly *Klebsiella pneumoniae* and *Escherichia coli*) [92], while *E. histolytica* is the main culprit in ALA. Additionally, PLA affects older male and female patients (generally in the 6th or 7th decade of life) with a history of diabetes or cholelithiasis, while ALA usually afflicts younger males in resource-limited settings [92]. Blood samples in PLA generally show elevated bilirubin with increased left-shift in white blood cells and hypoalbuminemia, while ALA demonstrates no left-shift, but increased bilirubin levels [88–91]. PLA also occurs in non-endemic settings more commonly and has no sex bias. Considering that PLA is the main differential for ALA, distinguishing between them is crucial for effective treatment and resolution of symptoms [44].

**Table 1:** Differences between ALA and PLA

Comparing amoebic liver abcess (ALA) and pyogenic liver abscess (PLA)	
Amoebic liver abscess (ALA)	Pyogenic liver abscess (PLA)
• Mainly monomicrobial with *E. histolytica* • Affects younger males in resource-limited settings [88] • Blood samples display no left-shift in white blood cells [88–90] • ALA more commonly affects males than females • Hyperbilirubinemia [91] • Clinical presentation pyrexia, right upper quadrant pain, hepatomegaly	• Polymicrobial with *Klebsiella pneumoniae* and *Escherichia coli* [92] • Affects older male and female patients with history of diabetes or cholelithiasis • Blood samples generally display elevated bilirubin with increased left-shift in white blood cells and hypoalbuminemia [88–90] • PLA also occurs in non-endemic settings more commonly and has no sex bias • Clinical presentation pyrexia, right upper quadrant pain, hepatomegaly

## Current diagnostic modalities in Sri Lanka

ALA is a clinical diagnosis in an endemic region such as SLK. It is supported by the typical radiological appearances presented in ultrasonography of the liver, which is an accessible, non-invasive imaging modality available at most state hospitals. Ultrasonography enables the treating clinician to determine the size, site, and type of abscess present. Typical liver imaging demonstrates a hypoechoic liver mass. With advancements in molecular biology, ELISA kits are being used for immune diagnosis of *E. histolytica* infection. For study purposes, PCR for amoeba is performed from aspirated pus, is also used to diagnose amoeba; however, the accessibility and cost are significant limiting factors, in utilizing these modalities.

The use of PCR and immunological/molecular profiling for *E. histolytica* is seldom used in SLK. Indeed, the first published report providing immunological and molecular evidence that *E. histolytica* as a significant cause of liver abscesses in northern Sri Lanka was in 2018 by Kannathasan and colleagues [48]. In this 3-year study, the authors employed nested PCR from DNA of 50 aspirated pus samples based on the serine-rich *E. histolytica* protein (SREHP) coding gene. The *E. histolytica* DNA sequence was observed in all samples. The authors also employed IgG ELISA using pus and serum samples from 346 patients and reported 99.7% of samples to be positive for IgG against *E. histolytica*, which is comparable to earlier studies [94, 95]. An earlier study by Khairnar et al. (2007) utilized nested PCR in 200 samples on various *Entamoeba spp.*, and concluded that it was a more sensitive diagnostic modality in the detection of *E. histolytica*, *Entamoeba moshkovskii* (*E. moshkovskii*), and *E. dispar*, compared with ELISA [29]. In another study, Dinoop et al. (2016) compared nested-multiplex, Taqman, and SYBR green real-time PCR as diagnostic modalities for ALA. They concluded that Taqman real-time PCR against the 18s rRNA was the most accurate test, demonstrating the highest positivity rate compared with the other two modalities. Dinoop et al. (2016) also demonstrated that real-time PCR can be utilized as a reliable diagnostic modality, compared with other conventional molecular methods [96].

Despite their limited utility in resource-limited settings such as SLK, serological methods such as PCR and ELISA have high specificity and sensitivity. However, a combination of serology with PCR/antigen detection remains the best approach in diagnosis, as this may increase the overall sensitivity and specificity of diagnosis [97]. A recent study by Wong et al. (2017) employed parallel ELISA using crude soluble antigen and excretory-secretory antigen for *E. histolytica* trophozoite detection in aspirated pus samples. They demonstrated that utilizing both assays improved the overall efficacy of amoebic serology compared with one assay alone [98]. Additionally, in the presence of imaging findings suggestive of ALA, confirmatory testing using serology and antigen detection should be commenced. This could involve stool microscopy or ELISA-based stool antigen detection. However, considering that ALA and *E. histolytica*-related colitis are unlikely to occur simultaneously, stool microscopy would generally be negative in this scenario. Additionally, stool microscopy cannot reasonably distinguish between *Entamoeba spp.*, and thus molecular testing of aspirated pus may be an alternative method [44, 99]. Nonetheless, the utilization of a combination of diagnostic modalities is particularly crucial in endemic settings, as patients may have been exposed to *E.histolytica* repeatedly, and thus may remain asymptomatic for years after infection [100]. This may confuse the treating clinician due to the inability to distinguish between past and current infection. However, while a shortcoming of serological tests is false positive, based on prior infection in an endemic area, the antibody response to the Gal/GalNAc lectin is not prolonged. In fact, Haque et al. (2000) demonstrated that ALA can be accurately (96%) diagnosed via antigen detection of the Gal/GalNAc lectin in patients who had not received metronidazole, in addition to being used as a test of cure [101]. Therefore, accurately selecting methods of diagnosis based on prior infectivity can aid in definitive diagnosis and an appropriate management plan [48].

## Radiological findings in amoebic liver abscess

Focal hepatic lesions of the liver have a broad differential diagnosis heavily dependent on the clinical context of the patient. Ultrasonography and computed tomography (CT) are by far the most utilized modalities, typically in that order, owing to availability and relatively low-cost. When combined, they can yield a confirmed diagnosis of ALA in over 90% of cases; however, triple-phase CT has higher sensitivity [61, 102]. Generally, radiological findings of liver abscesses typically consist of circumscribed round or ovoid hypoechoic masses which may be commonly solitary or less commonly multifocal. The central or internal contents are typically of slightly higher complexity than simple fluid and it is common to find a peripheral rim or capsule of enhancement on post-contrast computed tomography assessment without internal enhancement.

The trophozoites access the liver from the gastrointestinal tract via the portal vein to initially form micro-abscesses which progress and coalesce to a single large lesion [103]. ALA typically demonstrate two imaging phases: pre-suppurative and suppurative. On CT, pre-suppurative consists of heterogeneous, hypodense, and irregularly contoured lesions that are difficult to differentiate from more aggressive lesions such as tumors. This is important when assessing the clinical context and formulating a differential diagnosis [61]. During the suppurative phase, the appearance becomes more classic for abscesses with coalescence, thin or thick peripheral rim of post-contrast enhancement, and a ‘target’ or ‘ring-enhancing’ appearance [61].

**Fig. 2:**
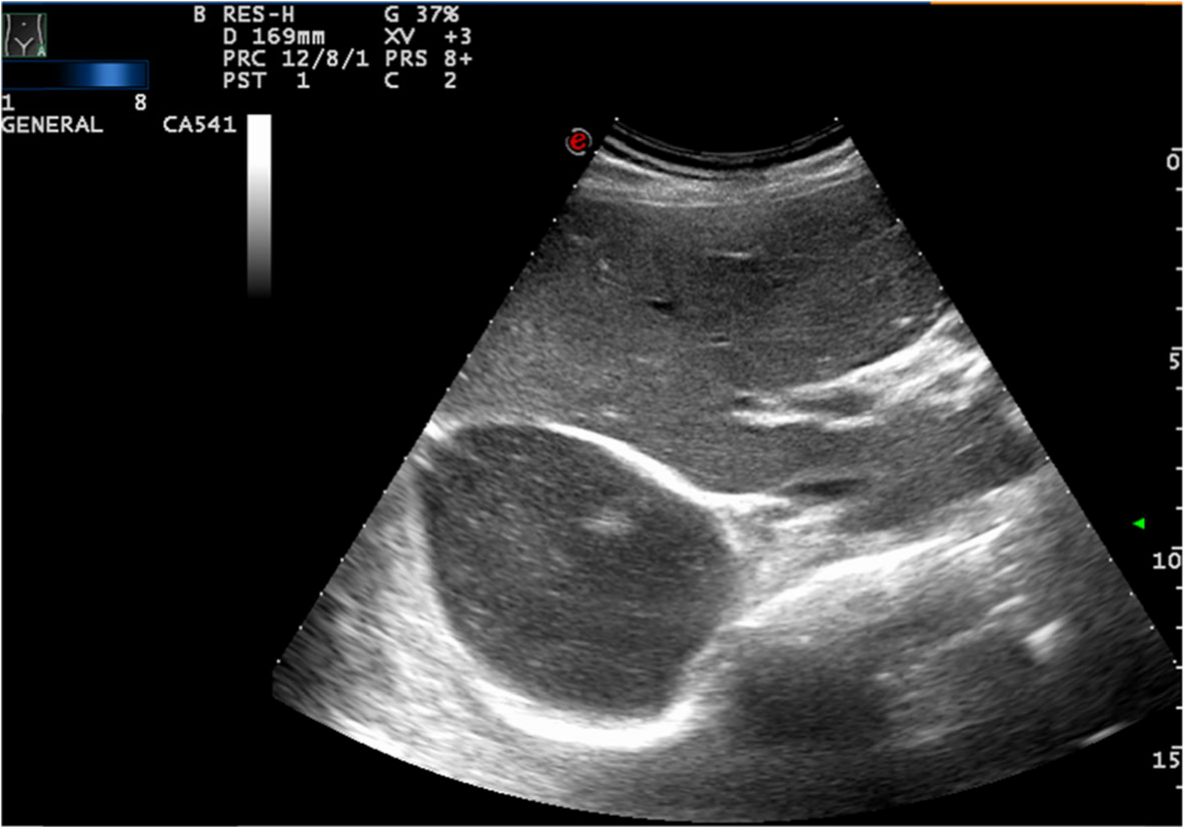
Ultrasonographic image of ALA in a male patient at Teaching Hospital Jaffna, demonstrating a solitary large right lobe liver abscess

When distinguishing between differential considerations, a solitary lesion within the right hepatic lobe ranging from 4 to 12 cm is more typical of ALA (Fig. 2); however, multifocality does not serve to rule out ALA [44]. Moreover, although ultrasonography is a common starting point for imaging in screening for or confirmation of ALA, a negative ultrasound study should be followed up with CT given differences in sensitivity, predominantly due to operator-dependency of ultrasonography [44]. The main differential consideration is for a pyogenic liver abscess given it is more common than ALA in nonendemic settings [104]. On a case-by-case basis, magnetic resonance imaging (MRI) can be considered for further characterization of the lesion or narrowing of the differential diagnosis, although the modality can be cost- and access-prohibitive in typical endemic regions. Classic appearances would be homogeneously low T1 and high T2. MR signal intensity of the lesion(s) with per-lesional increased T2 signal edema in approximately half the lesions [105]. Generally, the combination of imaging, clinical features, and serology allow for diagnosis in almost all cases with aspiration or drainage of an uncomplicated ALA [62] .

## Current approaches to management in Sri Lanka

Current approaches to treatment in SLK include amebicidal tissue-active agents, such as oral metronidazole therapy (500–750 mg three times orally for approximately 7–10 days), which has been shown to be the drug of choice in 85% of cases in SLK [5, 106]. This is largely owing to the fact that it is the only amebicidal drug available in the state sector. Other amebicidal agents that can be used to treat ALA include ornidazole, tinidazole, nitazoxanide, and chloroquine [107, 108]. Patients are expected to commence on luminal medications to eradicate intestinal colonization in the cecum, such as paromomycin (25–30 mg/kg orally three times daily for 7 days) [63, 109]. While there is strong evidence demonstrating that monotherapy is often inadequate in invasive disease, it is not standard practice in SLK, due to being in a resource-limited setting [110]. Other treatment approaches such as ultrasonography-guided drainage of ALA remain controversial, with no difference in mortality rates between medical therapy and aspiration of abscesses in uncomplicated ALA [49, 52]. A Cochrane review concluded that in uncomplicated ALA, aspiration combined with metronidazole compared with metronidazole alone as a treatment modality cannot be supported or contraindicated [106]. A more recent study by Chandak et al. (2019) demonstrated both procedures to be equally effective in the treatment of liver abscesses. The decision to aspirate at the discretion of the treating clinician. In practice, large abscesses (> 5 cm), left lobe abscess, and an abscess with a thin rim of liver parenchyma around it (peripheral abscess) should undergo percutaneous needle aspiration (PNA) owing to the risk of rupture into the pericardium, pleural cavity, and peritoneum which results in devastating consequences, such as peritonitis and septic shock [44]. The former occurs in approximately 7% of all cases [111]. In these situations, surgery is necessary and involves a transperitoneal or posterior transpleural approach [112]. Non-resolving pyrexia and persistent symptoms even after 72 h of initiating tissue amebicides occur indicating poor response to treatment and are also an indication for drainage [113, 114]. Generally, about 15% of all cases are refractory to medical therapy, and 20% of ALA cases may be further exacerbated by superimposed secondary bacterial infection [115, 116].

**Fig. 3:**
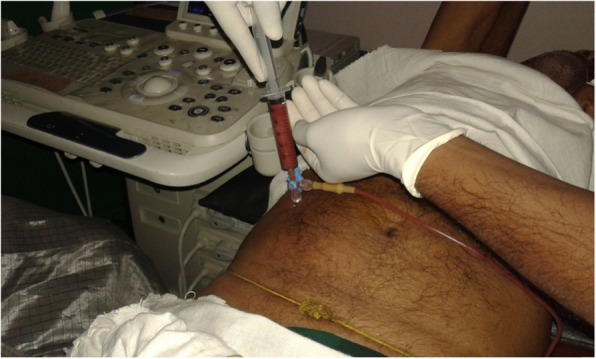
Therapeutic ultrasonographic-guided percutaneous aspiration of ALA in the right-upper quadrant at THJ—a relatively safe bedside procedure

With advances in imaging modalities, minimally invasive ultrasound-guided procedures are utilized, such as ultrasonography-guided percutaneous drainage (Fig. 3), where success rates have been reported to range from 70% to 100% [117]. Drainage of the abscess can either be performed with PNA or via pigtail catheter drainage (PCD). The latter has been proven to be a safe technique for both ALA and PLA and has also been shown to result in early relief patients [118]. In comparing these two modalities, pigtail catheter drainage has been shown to be a better option in patients with large abscesses. Despite no differences in mortality, catheter drainage results in faster clinical relief and a shorter duration of parenteral antibiotics [119, 120]. In an Indian study of 190 patients, of which 95 were treated with PCD, mean hospital stay was shorter, clinical improvement and reduction in cavity size by 50% were faster in patients treated with PCD compared with PNA. PCD has also been shown to be more effective in the management of PLA, compared with PNA [121]. Indeed, a systematic review of 5 randomized controlled trials of 306 patients reported a 29% higher success rate with PCD compared with PNA group in patients treated for ALA [relative risk (RR) 0.81, 95% confidence interval (CI) 0.66–0.99; *p* = 0.04] [122]. PCD results in more rapid improvement of symptoms as it provides continuous catheterization avoiding re-accumulation of pus and providing more frequent evacuation [117]. However, the main drawbacks of PCD are its higher cost and invasive nature, and a greater likelihood of complications, such as catheter displacement, septicemia, and hemorrhage [123]. Nevertheless, an aforementioned systematic review concluded no significant differences in complications between PCD and PNA [122].

Overall, ALA responds well to treatment with cure rates of > 90% reported with medical therapy. With appropriate intervention, symptoms typically improve in 3–4 days [124]. Poor prognostic indicators of ALA include hypoalbuminemia (serum albumin < 2.0 g/dL), size and number of abscesses, encephalopathy, and bilirubin levels greater than 3.5 g/dL, which have all been shown to be independent predictors of mortality [59] .

## Barriers to elimination

Eradication of ALA has been particularly difficult in the northern peninsula of SLK and can be attributed to a multitude of factors. Specifically, over 30 years of prevailed internal political unrest has induced a substantial toll on the healthcare system in the region, as the north and east were the primary conflict zones. The armed conflict adversely affected the projected public health trajectory, resulting in overall worse health outcomes post-war [125], and despite post-conflict healthcare system reconstruction being underway, unmet needs remain. These include water and sanitation systems, access to healthcare facilities, general health awareness programs, lack of basic health knowledge, human resources for health, and inadequate infrastructure. Nagai and colleagues (2007) identified the aforementioned disparities from self-administered questionnaires to healthcare providers and inhabitants of the northern region [126]. As an example, despite the fact that *E. histolytica* transmission can be reduced markedly with the provision of safe drinking water and sanitation, safe drinking water and adequate latrine facilities deteriorated during the armed conflict and have not completely recovered [126]. In fact, the development of adequate latrine facilities in the north has lagged behind 20 years compared with national averages [126]. Thus, the primary barrier to eradication continues to be poor sanitation and hygiene in the northern region, which has been slow in development compared with other parts of the island post-conflict.

The persistence of ALA in the northern peninsula despite reduced prevalence rates in other parts of the island has been deemed a public health failure [11]. Therefore, empowering residents through implementing health awareness programs (education about harms of regular alcohol consumption [and in particular, the indigenously brewed beverages], mass media campaigns) and improving infrastructure (improving access to healthcare facilities, adequate latrine facilities, improving conditions at toddy taverns) are all crucial to formulate effective elimination strategies for *E. histolytica*-related ALA infections.

## Prevention and future directions

At present, no vaccine exists to prevent *E. histolytica*-induced ALA. Thus, primary preventive efforts should emphasize the importance of improving sanitation, the provision of safe drinking water, and public health campaigns to promote hygienic practices (i.e., hand-washing, provision of adequate supplies, improving latrine facilities, promoting safe consumption of alcoholic beverages as harm reduction) and food safety [12, 127]. One example is the ‘five keys to safer food’ (i.e., utilization of clean water and utensils when cooking, separation of raw and cooked foods, ensuring foods are kept at the appropriate temperature and are cooked thoroughly) [128]. Increasing drinking-water hygiene standards in areas endemic to *E. histolytica* is recommended. The WHO Guidelines for Drinking-Water Quality shows that *E. histolytica* has a high resistance to traditional low-dose chlorination practices and transmission of *E. histolytica* through potable water sources is common in the tropical regions [129]. The WHO’s guidelines recommend that drinking water be prevented from contamination by human waste and that adequate protection is provided during the transportation of potable water supplies. The boiling of drinking water for 1 min or the addition of iodine to drinking water supplies is highly recommended to sanitize water sources of *E. histolytica* cysts prior to human consumption. Water Safety Plans (WSPs) are a WHO framework that provide a risk management strategy for drinking water supplies and provide a systematic approach to ensure that the safety and quality of the drinking water meets WHO standards. WSPs are covered in WHO’s Guidelines for Drinking-Water Quality and if utilized properly, will provide the policies and framework necessary to implement the WHO recommendations to ensure quality drinking water [129].

Another preventive strategy can be employed amongst child-rearing women. Expectant mothers and those with infants should be taught the importance of breastfeeding (i.e., breastfeed exclusively for the first 6 months of life and up to 2 years and beyond), as it is an effective way to protect them from acquiring diarrheal disease [12]. This is important as *E. histolytica* has been listed as one of the top-15 causes of diarrhea in the first 2 years of life [2, 127].

Educating patients about the harms of regular alcohol consumption is also warranted. There appears to be a lack of understanding amongst the population about regular consumption of alcohol and liver damage [67]. Additionally, there is also a general lack of knowledge amongst patients about ALA, the causative agent, risk factors, and the adoption of preventive measures [6]. The World Health Assembly’s adopted resolution EB126.R11, a global strategy to reduce the harmful use of alcohol, has undergone new adaptations, including expanding the evidence base such that it applies to low- and middle-income countries. In addition to this, the WHO has adopted scientific support for the interventions in its new global strategy, including to increase the capacity of health and social welfare systems to deliver treatment and early intervention, limits on alcohol availability, restrictions on alcohol marketing, and pricing policies to discourage alcohol consumption [130].

Strategies to increase awareness such as mass media campaigns and community-level interventions are necessary, particularly to those who are at risk, in order to eradicate the disease from the island. These could include education about safe limits of alcohol consumption, as frequency and type of alcohol consumed have been shown to be risk factors for ALA [6], as well as education on safe brewing practices of indigenous beverages as a harm reduction strategy.

## Conclusion

*E. histolytica* is a public health problem in northern SLK, owing to poor sanitation and poor hygienic practices, particularly in the production of indigenous alchoholic beverages. Middle-aged men with a history of alcohol consumption are particularly at high risk, which is exacerbated by the lack of knowledge and attitudes about ALA, its route of transmission, and general neglect towards practicing good hygiene in toddy taverns. As a preventable illness, this imposes a significant burden on the healthcare system and substantial opportunity for improvement still exists to address this healthcare disparity.

## Data Availability

Not applicable.

## Abbreviations

ALA: Amoebic liver abscess
CP: Cysteine proteases
CT: Computed tomography
*E. dispar*: *Entamoeba dispar*
*E. histolytica*: *Entamoeba histolytica*
*E. moshkovskii*: *Entamoeba moshkovskii*
ELISA: Enzyme-linked immunosorbent assay
Gal/GalNAc: Galactose and *N*-acetyl-d-galactosamine
IBD: Inflammatory bowel disease
PCD: Pigtail catheter drainage
PCR: Polymerase chain reaction
PLA: Pyogenic liver abscess
PNA: Percutaneous needle aspiration
rRNA: Ribosomal ribonucleic acid
SES: Socioeconomic status
SLK: Sri Lanka
THJ: Teaching Hospital Jaffna
WHO: World Health Organization
WSPs: Water Safety Plans
